# Bone - a casualty of ICU survival?

**DOI:** 10.1186/s13054-015-0977-7

**Published:** 2015-06-18

**Authors:** Karin Amrein, Astrid Fahrleitner-Pammer, Hans Peter Dimai

**Affiliations:** Department of Internal Medicine, Division of Endocrinology and Metabolism, Medical University of Graz, Auenbruggerplatz 15, A-8036 Graz, Austria

We read with great interest the pilot study on fracture risk in 46 critically ill patients by Rawal and colleagues [1]. Bone mineral density (BMD) was assessed with portable calcaneal dual X-ray absorptiometry (DXA) on days 1 and 10. The authors found no overall change in BMD, but increased fracture risk in the subgroup with 'ARDS' (acute respiratory distress syndrome) (n = 34). There are several serious methodological problems: it is unclear if the group assignment is valid as only one of the four Berlin definition criteria [2] for the diagnosis of ARDS is given: oxygenation (no information on timing, chest imaging and origin of the edema). Although peripheral DXA devices are certainly an interesting option for BMD in critically ill and other patients [3], precision errors supplied by the manufacturer should not be used. In fact, each center must determine its (own) precision error and least significant change, which is also operator dependent [4]. The manufacturer stated a 0.9 % coefficient of variation, which is not necessarily applicable to the study setting where it remains unclear how the measurements were performed. This could be an important limitation. Hence, it is unlikely that a 2 % BMD change within such a short time frame as described would reflect anything else other than random variability. Lastly, it is also unclear what the described statistical difference refers to - it should be noted that the smaller subgroup (n = 12) numerically increased their BMD. Undoubtedly, fracture risk following critical illness is a very important topic that requires further high-quality studies [5].

## Abbreviations

ARDS: Acute respiratory distress syndrome
BMD: Bone mineral density
DXA: Dual energy X-ray absorptiometry
